# Demonstration of electron beam laser excitation in the UV range using a GaN/AlGaN multiquantum well active layer

**DOI:** 10.1038/s41598-017-03151-8

**Published:** 2017-06-07

**Authors:** Takafumi Hayashi, Yuta Kawase, Noriaki Nagata, Takashi Senga, Sho Iwayama, Motoaki Iwaya, Tetsuya Takeuchi, Satoshi Kamiyama, Isamu Akasaki, Takahiro Matsumoto

**Affiliations:** 1 Faculty of Science and Technology, Meijo University, Nagoya, 468-8502 Japan; 2 Akasaki Research Center, Nagoya University, Nagoya, 464-8603 Japan; 3 Graduate School of Design & Architecture, Nagoya City University, Nagoya, 464-0083 Japan; 4 Graduate School of Medical Sciences, Nagoya City University, Nagoya, 464-0083 Japan

## Abstract

This study investigated electron beam laser excitation in the UV region using a GaN/AlGaN multiquantum well (MQW) active layer. Laser emission was observed when the GaN/AlGaN MQW was excited by an electron beam, with a wavelength of approximately 353 nm and a threshold power density of 230 kW/cm^2^. A comparison of optical pumping and electron beam pumping demonstrated that the rate of generation of electron-hole pairs when using electron beam excitation was approximately one quarter that of light excitation.

## Introduction

A number of recent breakthroughs have allowed the development of group III nitride semiconductor-based blue, green, and white light-emitting diodes (LEDs)^1–3^. These developments include the growth of high-quality GaN on sapphire, using a low-temperature (LT)-deposited buffer layer^4^, and the realization of conductivity control for nitrides^1, 5^. These technologies have also been used to create high-power violet laser diodes^6, 7^.

The expansion of the laser emission wavelength is a major research focus in the development of nitride semiconductor-based laser diodes. In the longer wavelength region, such diodes have been developed in both the blue^8^ and green regions^9, 10^. This has been achieved through optimization of the conditions under which the crystals are grown, and of the structure of the devices. At shorter wavelengths, however, nitride semiconductor-based laser diodes have only been achieved in the UV-A region^11–15^. To the best of our knowledge, the shortest wavelength reported for an AlGaN-based UV edge emitting laser is 326 nm^16^. Many issues remain to be resolved if the wavelength at which laser diodes can operate is to be decreased.

To realize laser oscillation, the material must exhibit optical gain, an optical resonator must be formed, and carrier injection must be realized. The key characteristics of AlGaN materials are their high optical gain and ability to form optical resonators. However, the development of UV laser diodes is being held back by the current lack of injection technologies that allow both a high hole concentration and low resistivity p-type AlGaN with a high AlN molar fraction to be realized^17^. In the case of AlGaN with a high AlN molar fraction, laser oscillation can be initiated using optical pumping^18, 19^. UV lasers with controllable wavelengths should therefore become possible if this problem can be resolved.

A promising approach to addressing this is the use of electron beam excitation. Existing nitride semiconductor-based lasers have been designed to achieve population inversion of the carrier and to oscillate in response to current injection. However, as noted above, it is difficult to achieve wavelengths shorter than 326 nm when using this method. However, the use of electron beam excitation renders the conductivity control of nitride semiconductors unnecessary. This should allow the wavelengths available for use with nitride semiconductor-based lasers to be extended deep into the UV region.

A number of studies have reported electron beam excitation of nitride semiconductors^20–22^. Both spontaneous UV light emission and visible laser light oscillation have been realized. However, electron beam excitation of laser oscillation in the UV region using nitride semiconductors has not yet been reported.

In this study, we investigated electron beam laser excitation in the UV region, using a nitride semiconductor device with a GaN/AlGaN multiquantum well (MQW) active layer.

## Methods

**Figure 1:**
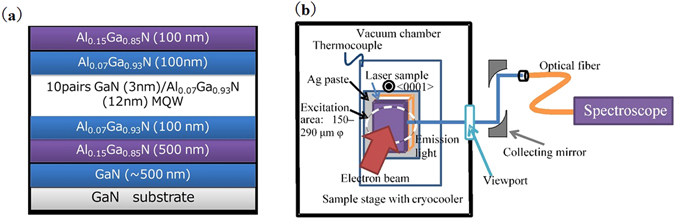
(**a**) Schematic view of the sample structure. (**b**) Exterior photograph of the electron beam excitation and measurement systems.

The sample used in the study was grown using metalorganic vapor-phase epitaxy. Figure 1(a) shows a schematic of the laser structure, which comprised a separate confinement heterostructure with a GaN/AlGaN MQW active layer grown on a c-plane freestanding GaN substrate. The optical confinement factor of this structure was about 6%. Trimethylaluminum, trimethylgallium, triethylgallium, and ammonia were used as the source gases. The stack comprised, in order, a 500-nm-thick homoepitaxial GaN layer, a 500-nm-thick Al_0.15_Ga_0.85_N cladding layer, a 100-nm-thick Al_0.07_Ga_0.93_N optical guide layer, 10 pairs of GaN (3 nm) and Al_0.07_Ga_0.93_N (12 nm) as the MQW active layer, a 100-nm-thick Al_0.07_Ga_0.93_N optical guide layer, and a 100-nm-thick Al_0.07_Ga_0.93_N cladding layer. The laser cavity was formed by a combination of Cl_2_ inductively coupled plasma etching and wet etching using the tetramethylammonium hydroxide aqua (~25 at%) method^23^. The laser sample had a cavity length of approximately 50 μm. Because the cavity length was short, no facet coating was applied.

Figure 1(b) gives a schematic view of the electron beam excitation and measurement systems. The laser sample was mounted on a cooling stage fitted with a cryocooler. The sample was mounted using Ag paste, and the chamber was then evacuated to approximately 1 × 10^−5^ Pa using a turbo molecular pump. An LaB_6_ electron beam gun was used as the excitation source, and the luminescence from the sample was determined from the acceleration voltage of the electron beam.

**Figure 2:**
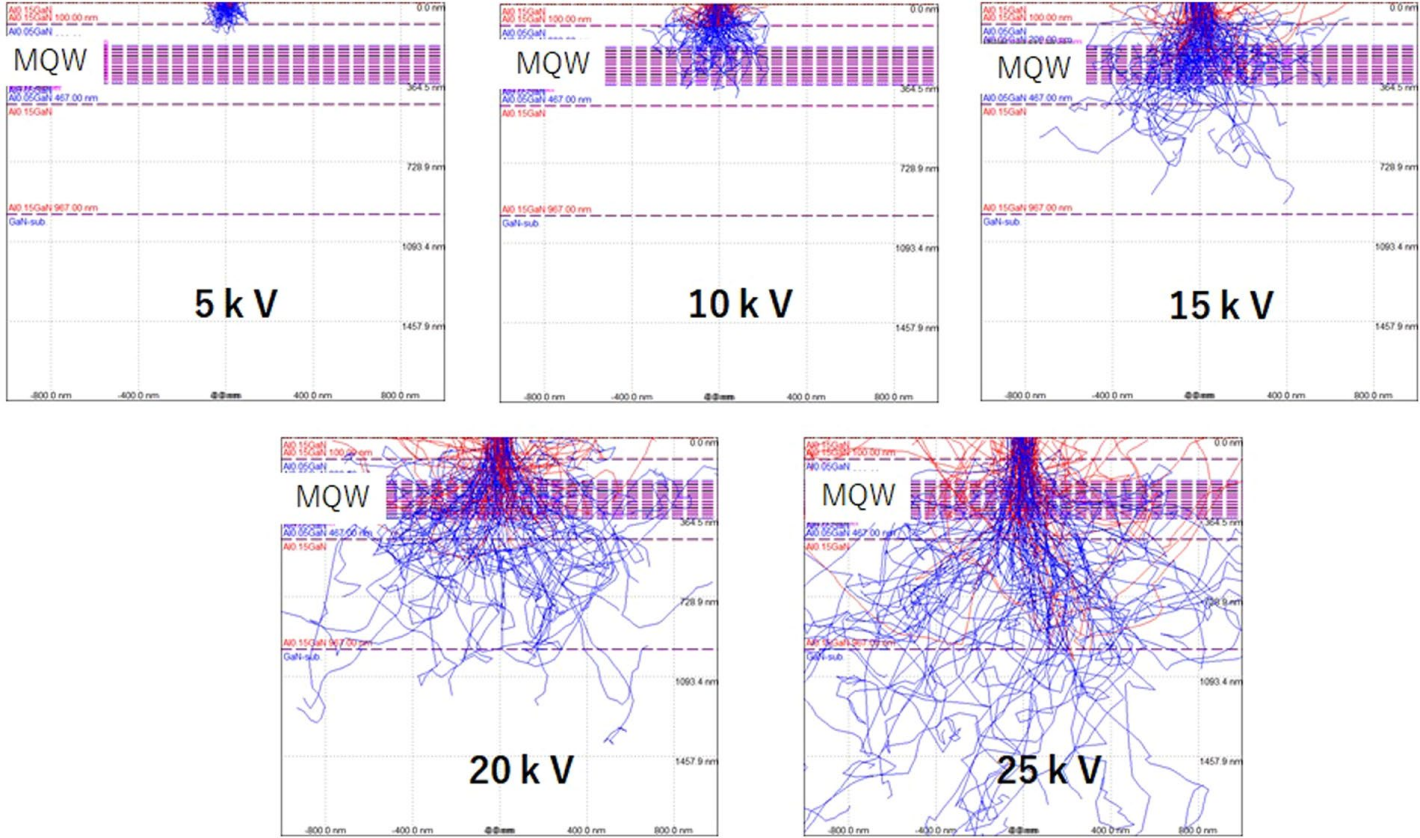
Trajectory of electron beam with accelerating voltage.

Before the experiment was run, a Monte Carlo simulation was conducted to identify the trajectory of the electron beam when the acceleration voltage was changed^20^. A CASINO ver.2 simulator was used. Figure 2 show the trajectory of the electron beam at acceleration voltages ranging from 5 to 25 kV, in increments of 5 kV. The blue and red lines in the figure show the trajectories of the primary and reflection electrons, respectively. Based on the results, the acceleration voltage was set at 15 kV. The electron beam current was measured using a Faraday cup and was varied across the range 0.02 to 5 mA. The portion of the sample irradiated by the electron beam was evaluated using a micro CCD image sensor. The excitation spot sizes of the electron beam were controlled to approximately 170 μm φ. The electron beam was set in pulse irradiation mode (pulse width 20 ns, cyclic frequency 3 MHz, and duty 6%). The sample was analyzed after cooling to approximately 107 K. The luminescence light emission from the sample edge was passed through a viewport and two collecting mirrors, and detected using a spectrometer with a CCD array (ANDOR SR-750-A-R and DU420A-UE; wavelength resolution ~0.04 nm) after passing through an optical fiber. The sample stage temperature was monitored using a thermocouple.

## Results and Discussion

**Figure 3:**
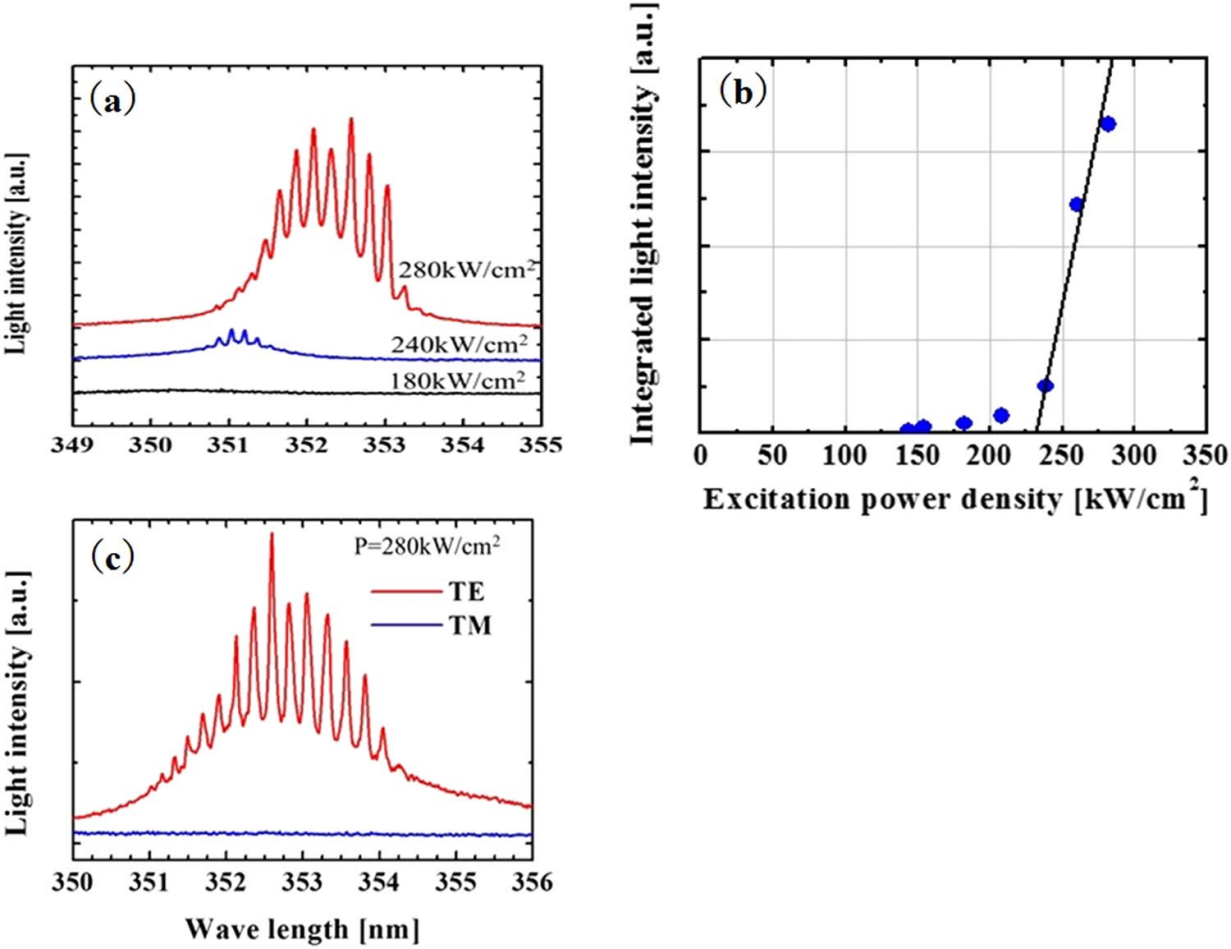
(**a**) Luminescence spectra from the GaN/AlGaN MQW active layer with excitation electron beam power levels of 180, 240, and 280 kW/cm^2^. (**b**) Integrated light intensity of the emission spectra as a function of the excitation electron power density. (**c**) Laser emission spectra of the TE mode and TM mode with excitation electron beam power level of 280 kW/cm^2^.

Figure 3(a) shows the luminescence spectra from the GaN(3 nm)/AlGaN(12 nm) MQW active layer after excitation by electron beams with power levels of 180, 240, and 280 kW/cm^2^. Figure 3(b) presents the integrated light intensity after deconvolution of the background level as a function of the excitation electron power density. At an electron beam power of 180 kW/cm^2^, spontaneous emission at 351 nm was observed. When the power was increased to 240 kW/cm^2^, a sharp emission was observed. The plot of the integrated light intensity as a function of the excitation electron power density clearly confirmed the presence of a threshold power density (*P*_th_) at approximately 230 kW/cm^2^. Above this threshold, the integrated light intensity increased linearly. Figure 3(c) shows the lasing spectrum passed by a polarizer (THORLABS GLB10), with a polarization feature at 280 kW/cm^2^. The lasing spectra and polarization feature suggested that the sample exhibited laser emission, stimulated by TE-polarization. Lasing emission was also observed at a wavelength of approximately 353 nm when the excitation power density was 280 kW/cm^2^.

**Figure 4:**
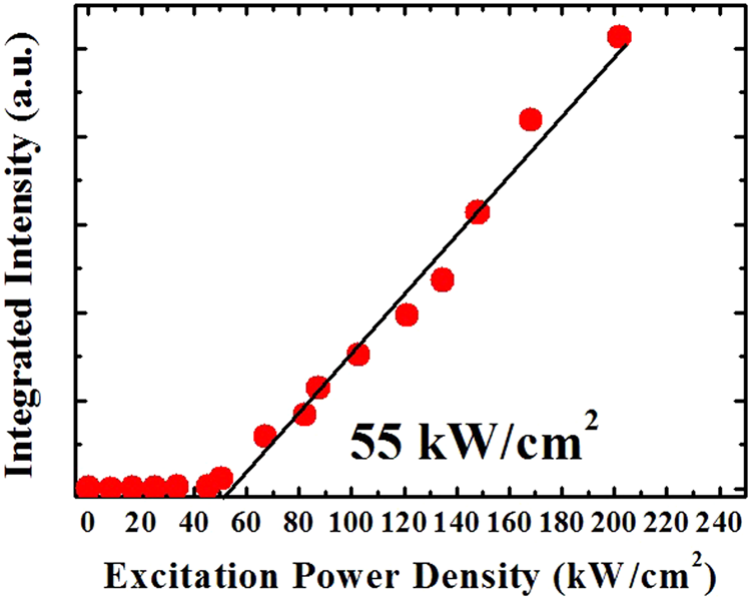
Integrated light intensity of the emission spectra as a function of the excitation optical power density.

We next compared the use of optical pumping and electron beam pumping. Figure 4 shows the excitation power dependence of the integrated light intensity by optical pumping using an Nd:YAG 4^th^ (λ = 266 nm) laser. The GaN/AlGaN MQW active layer produced laser oscillation at approximately 55 kW/cm^2^ when excited by the Nd:YAG laser. As noted above, the threshold power density (*P*_th_) was 230 kW/cm^2^ when using electron beam excitation. Laser oscillation by electron beam excitation was found to require four times the injection power of optical excitation. Since the same excitation carrier concentration is required for laser oscillation, this suggests that the generation rate of electron-hole pairs by electron beam excitation is approximately one quarter that of light excitation.

## Conclusion

In conclusion, this study investigated electron beam laser excitation in the UV region, using a GaN/AlGaN MQW active layer. The wavelength was found to be approximately 353 nm and the threshold power density 230 kW/cm^2^. We also compared the use of optical pumping and electron beam pumping. The rate of generation of electron-hole pairs when using electron beam excitation was approximately one quarter that of light excitation. This technology provides a means of addressing the problems associated with the current generation of injection systems. It offers a potential solution to the development of AlGaN-based UV lasers, and should contribute to the expansion of nitride semiconductor UV photonics.
